# Capturing hidden regulation based on noise change of gene expression level from single cell RNA-seq in yeast

**DOI:** 10.1038/s41598-021-01558-y

**Published:** 2021-11-19

**Authors:** Thoma Itoh, Takashi Makino

**Affiliations:** 1 Department of Biology, Faculty of Science, Tohoku University, 6-3, Aramaki Aza Aoba, Aoba-ku, Sendai, 980-8578 Japan; 2 Graduate School of Life Sciences, Tohoku University, 6-3, Aramaki Aza Aoba, Aoba-ku, Sendai, 980-8578 Japan

**Keywords:** Gene regulatory networks, Genome informatics

## Abstract

Recent progress in high throughput single cell RNA-seq (scRNA-seq) has activated the development of data-driven inferring methods of gene regulatory networks. Most network estimations assume that perturbations produce downstream effects. However, the effects of gene perturbations are sometimes compensated by a gene with redundant functionality (functional compensation). In order to avoid functional compensation, previous studies constructed double gene deletions, but its vast nature of gene combinations was not suitable for comprehensive network estimation. We hypothesized that functional compensation may emerge as a noise change without mean change (noise-only change) due to varying physical properties and strong compensation effects. Here, we show compensated interactions, which are not detected by mean change, are captured by noise-only change quantified from scRNA-seq. We investigated whether noise-only change genes caused by a single deletion of STP1 and STP2, which have strong functional compensation, are enriched in redundantly regulated genes. As a result, noise-only change genes are enriched in their redundantly regulated genes. Furthermore, novel downstream genes detected from noise change are enriched in “transport”, which is related to known downstream genes. Herein, we suggest the noise difference comparison has the potential to be applied as a new strategy for network estimation that capture even compensated interaction.

## Introduction

Cells are controlled and maintained by proteins translated from thousands of genes. Genes involved in cellular function interact with each other to exert their actions in cells^1^. To understand the regulatory mechanisms of gene expression, the regulatory relationships among genes have been closely examined^2^. Gene regulatory networks (GRNs) are directed graphs showing regulatory relationships of transcription factors (TFs) and their target genes. Since the GRN plays an important role in connecting genes and phenotypes, uncovering interactions contributes to understanding diverse biological phenomena. In recent years, a vast amount of expression data has rapidly accumulated through the development of high-throughput RNA-seq, resulting in data-driven GRN estimation methods, which have drawn much attention. Most GRN estimation methods are based on the assumption that perturbations in a specific gene affects downstream genes^3^. Capturing changes in expression levels due to the deletion of a specific gene effectively estimates molecules downstream of the deleted gene. However, the actual GRN is more complex, and may not be estimated with this simple assumption. Hundreds of genes downstream of *STP2*, which is a TF, have been validated by experimental approaches. Interestingly, most were detected from the double deletion of *STP1* and *STP2*^4,5^, and did not show a change in the mean expression level when only *STP2* was deleted^6^. This phenomenon would be explained by functional compensation of a *STP2* paralog. Since the lost function, upon *STP2* deletion, was compensated by *STP1*, which is the functionally redundant gene with *STP2*, the downstream genes of *STP2* are not affected by the *STP2* deletion^7^, resulting in no change in the expression levels of STP2 downstream genes.

**Figure 1:**
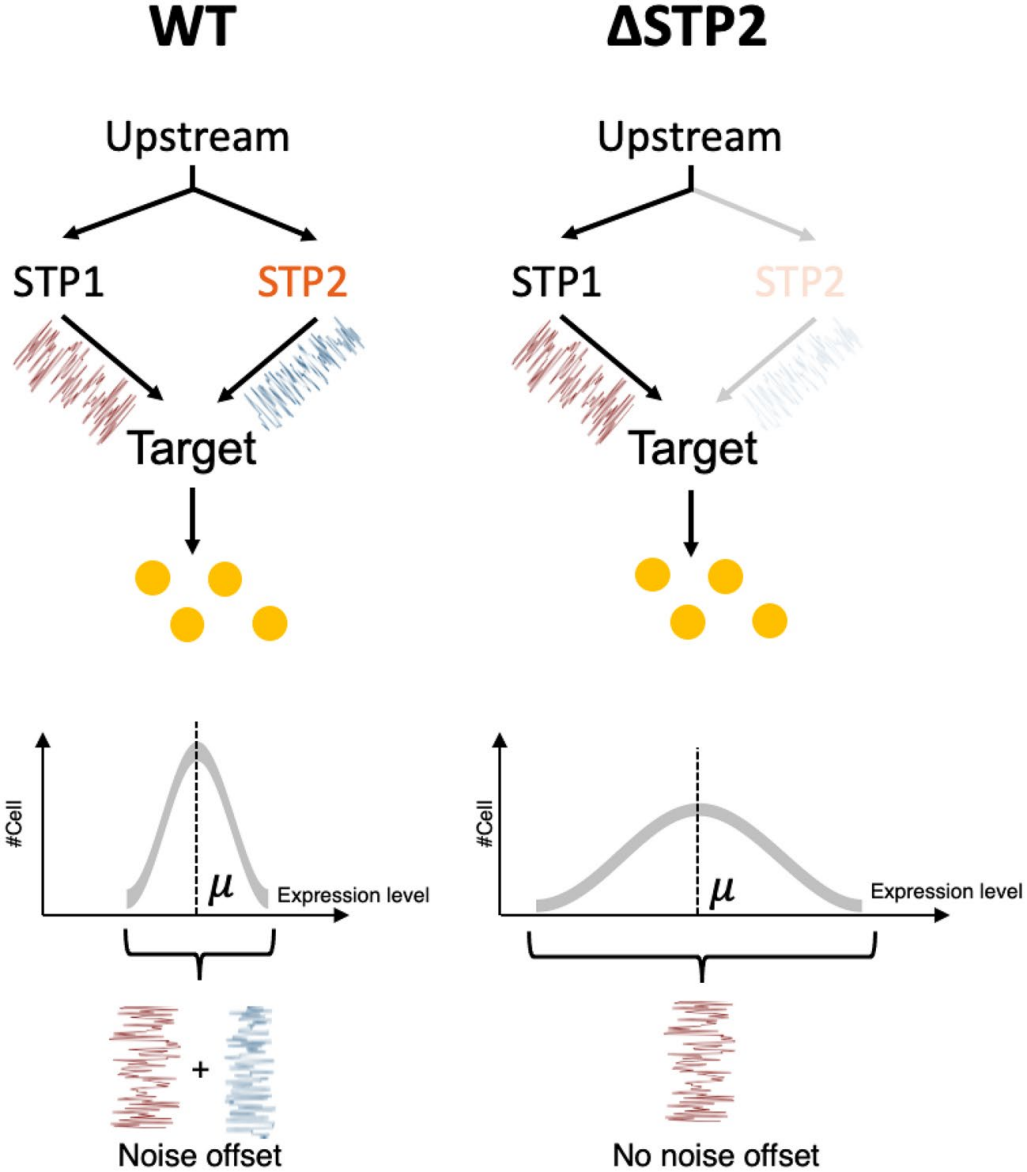
Noise difference detects functional compensation. STP1 and STP2 redundant pathway (Left: Wildtype, Right: STP2 deletion mutant). The lack of STP2 is compensated by STP1, resulting in no change in the mean expression level of downstream genes (orange circles). However, expression noise that propagated from upstream changes differ due to changes in physical characteristics of upstream genes. An example of change of distribution is shown at the bottom. The vertical axis represents the number of cells and the horizontal axis shows the expression level of the target gene. Whereas the variance was small because of the noise offset by the redundant pathway in the WT, the variance became large due to the lack of noise offset in ΔSTP2. Mean remains constant by the functional compensation effect. Note that whether the noise becomes large or small upon deletion is not evident, and that the direction of change is not an essential issue in this study.

While functional compensation provides robustness to perturbations in the GRN, it hinders estimation of downstream genes. To identify downstream genes that are not affected by upstream perturbations due to functional compensation, we focused on a change in expression noise by gene deletion. Expression noise is the stochastic variation in gene expression quantified by variance excluding the contribution of the mean in a clonal population^8^. Since expression noise is affected by the transcription or translational efficiency characterized by the physical property of a sequence^9^, even functionally redundant gene pairs may show different expression noise. Furthermore, since it has been reported that expression noise propagates from upstream genes, the expression noise altered by compensation of redundant genes is expected to propagate to downstream genes and alter the expression noise of downstream genes^9^. Based on these previous reports, we hypothesize that noise change can be observed downstream of functionally compensated genes due to the physical change in upstream genes (Fig. 1).

**Figure 2:**
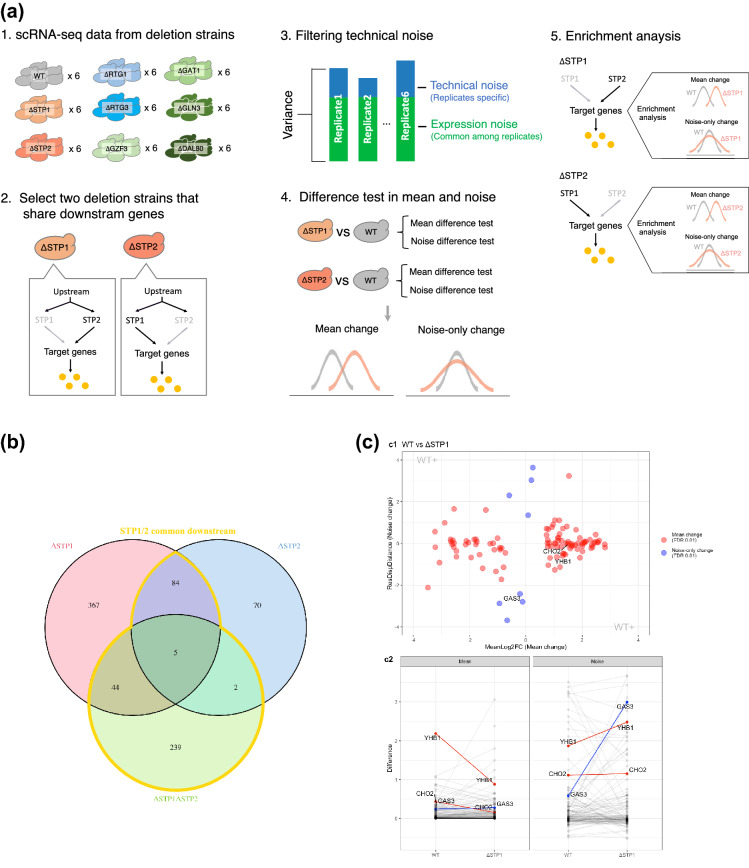
Schematic workflow and data processing. (**a**) Schematic workflow. 1) scRNA-seq data from wildtype and deletion strains. Each strain has 6 replicates that were separately constructed. 2) We selected two deletion strains that share a common downstream gene. Although STP1/2 are depicted, *RTG1/2* and GATA family (*GZF3, GAT1, GLN3,* and *DAL80*) were selected as pair (group) for the same reason. 3) Technical noise is eliminated by decomposing the total variance to technical noise and expression noise using the hierarchical Bayesian model. Briefly, common variance among replicates is recognized as expression noise, and replicate specific variance is recognized as technical noise. 4) Comparison between wildtype and the interest strain regarding mean and noise. Further, we derived the noise-only change genes that show significant noise change without mean change. An example of distribution change is shown at the bottom. 5) Investigation of the enrichment of mean change or noise-only change genes into the downstream genes shared by the pair genes (*STP1/2*, in this example). (**b**) Venn diagram of *STP1/2* downstream genes. The pink and blue areas represent genes that showed mean change in the single deletion, ΔSTP1 (500) and ΔSTP2 (161) respectively. The green area represents genes that showed mean change in the double deletion ΔSTP1ΔSTP2 (290). The area circled in yellow indicates the downstream genes shared by *STP1/2* (374). (**c**) Comparison of mean and noise change genes in WT vs ΔSTP1. c1) x-axis: mean expression change measured by log2 fold change (Mean Log2FC). y-axis: expression noise change measured by the distance of residual over-dispersion (ResDispDistance), which is the noise difference correcting the mean dependency. The red dots represent genes with at least mean change (*e.g., CHO2, YHB1*). The blue points represent genes with noise-only change which is the noise change without mean change (*e.g., GAS3*). c2) Left: mean expression level transition. Right: expression noise transition. *YHB1* and *CHO2* are the mean change genes and are showed by the red line. *YHB1* shows both mean change and noise change. *CHO2* shows only mean change without noise change. *GAS3* is the noise-only change gene and is showed by the blue line. Whereas *GAS3* showed significant noise change, mean expression was not changed.Schematic workflow for enrichment analysis of mean change genes and noise-only change genes to the target.

Notably, not all noise changes imply functional compensation, as noise change reflects changes in physical properties, such as upstream translational efficiency, and does not necessarily indicate functional compensation. If functional compensation occurs upstream, the genes must show noise change without mean change (noise-only change), due to changes in physical properties and compensation effects on the expression level. Therefore, we focused on genes that show noise-only change and investigated whether they are enriched in the common downstream genes of redundant gene pairs, such as *STP*1/2 (Fig. 2a).

In this study, we aimed to estimate mean-change genes and noise-change genes in yeast using scRNA-seq data by comparing expression patterns in a single deletion mutant (ΔSTP1, ΔSTP2, ΔRTG1, ΔRTG3, ΔGLN3, ΔGAT1, ΔGZF3, and $\Delta$DAL80; Table S1) with those in wildtype. Furthermore, we investigated enrichment of estimated genes to the downstream genes shared by a gene group, such as paralogous pair or protein complex members (Fig. 2a).

## Results and discussion

### Redundantly regulated target; *STP1* and *STP2*

*STP1* and *STP2* are a duplicated pair of genes that arose from whole genome duplication (ohnologs), and thus share many downstream genes. Since it has been reported that *STP1* and *STP2* have redundant functions^7^, those common targets are likely to be redundantly regulated. Regarding the downstream genes shared by *STP1/2* (*STP1* and *STP2*), the proportion of genes that show mean expression change in the ΔSTP1 and ΔSTP2 was 36% (133/374) and 24% (91/374), respectively. The remaining genes did not show mean expression change in the single deletion, which suggests functional compensation^4–6^ (Fig. 2b). Based on our hypothesis, those redundantly regulated genes can be detected by noise-only change, even in the single deletion of *STP1* or *STP2*.

First, we estimated mean-change genes and noise-change genes using the BASiCS R package. Mean and noise were inferred by comparing the gene expression of WT with that of the interest strain based on the hierarchical Bayesian model proposed in previous studies^10,11^. The relationship between inferred mean change and noise change is shown in Fig. 2c-1, and the transition of the inferred mean and noise is shown in Fig. 2c-2. From those relationships, we extracted noise-only change genes (Fig. 2c-1, blue dots) and mean-change genes (Fig. 2c-1, red dots).

**Figure 3:**
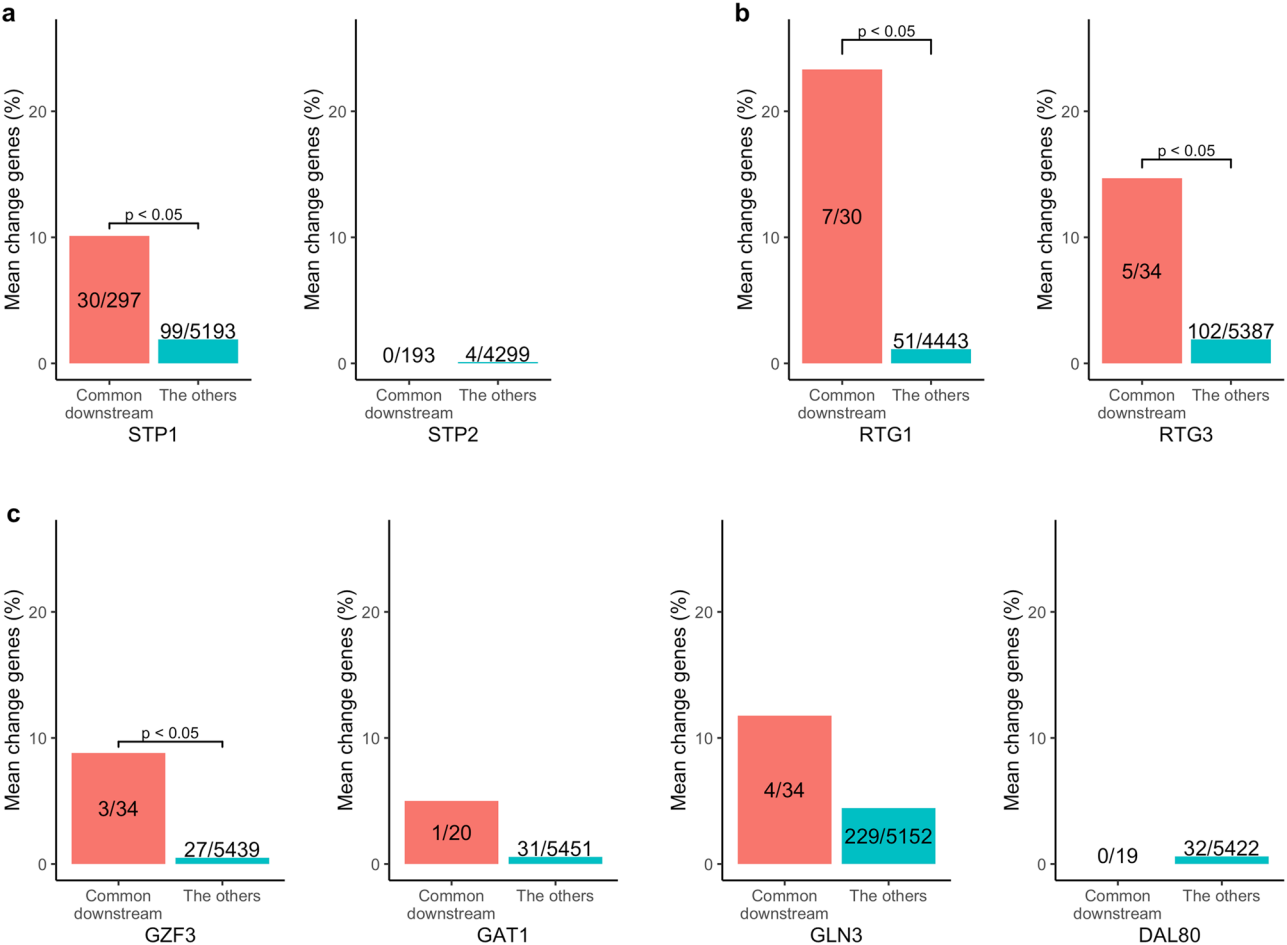
Ratio of mean change genes in known downstream genes shared by homologous groups. Bars show the proportion of mean change genes in the two categories labeled in the x-axis. Common downstream; the reported downstream genes that are shared by the gene groups in which the deleted gene belongs. The others; the other genes in the right category. Bars indicate the ratio of mean change genes in each category. Note that the numbers of common downstream genes (shown on the red bar) do not coincide (even within the group) because non expressed genes in each strain are excluded. (**a**) Redundant gene group; *STP1* and *STP2*. (**b**) Non redundant gene group; *RTG1* and *RTG3*. (**c**) Possibly redundant paralogous group; GATA family (*DAL80*, *GAT1*, *GLN3*, and *GZF3*). The *p*-values above each bar were calculated using Fisher’s exact test.

**Figure 4:**
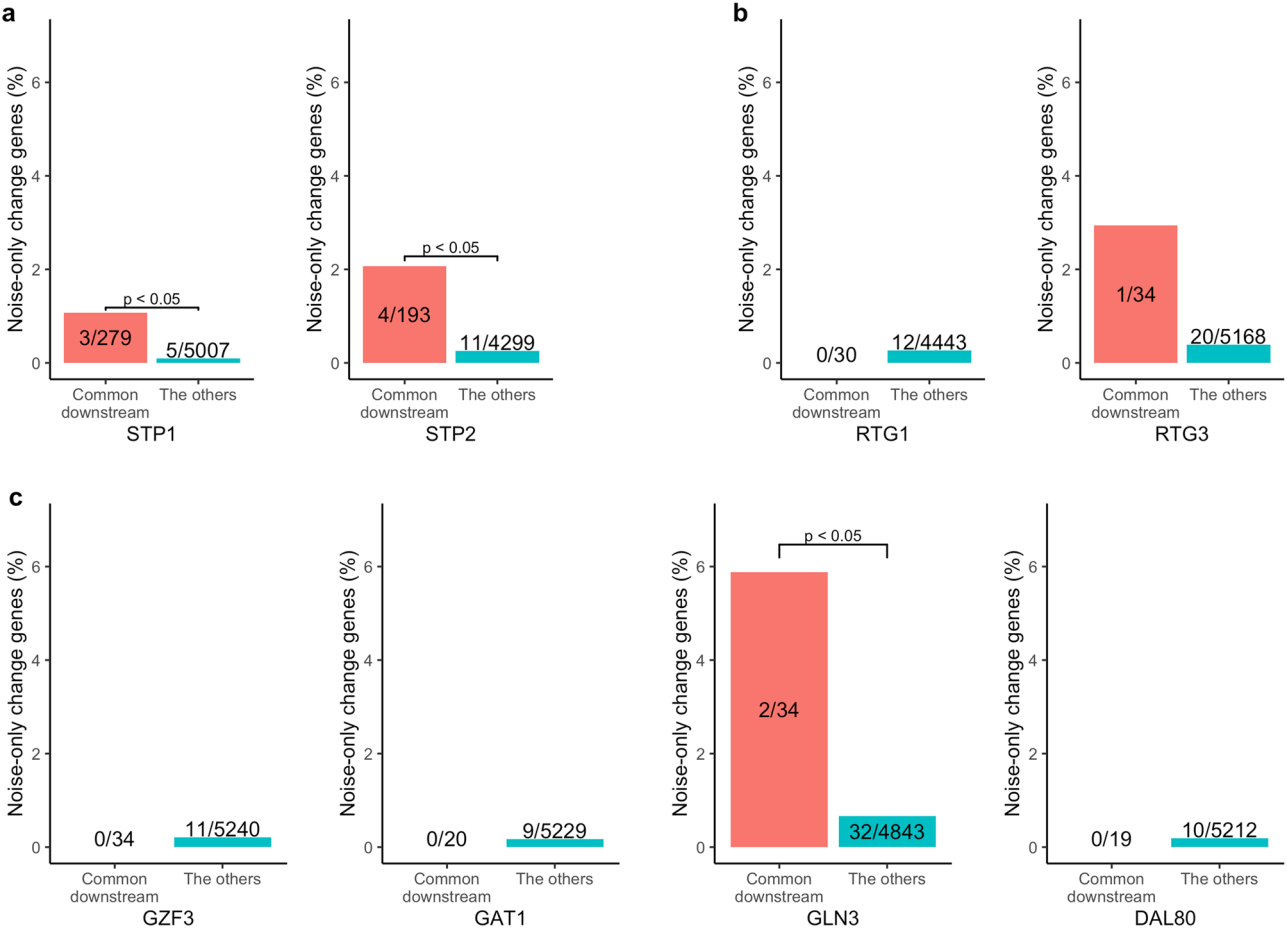
Ratio of noise-only change genes in known downstream genes shared by homologous groups. Bars show the proportion of noise-only change genes in the two categories labeled in the x-axis. Common downstream; the reported downstream genes that are shared by the gene groups in which the deleted gene belongs. The others; the other genes in the right category. Bars show the ratio of noise-only change genes in each category. Note that the numbers of common downstream genes (shown on the red bar) do not coincide (even within group) because non expressed genes in each strain are excluded. (**a**) Redundant gene group; *STP1* and *STP2*. (**b**) Non redundant gene group; *RTG1* and *RTG3*. (**c**) Possibly redundant paralogous group; GATA family (*DAL80*, *GAT1*, *GLN3*, and *GZF3*). The *p*-values above each bar were calculated using Fisher’s exact test.

We examined whether mean-change genes or noise-only change genes in ΔSTP1 and ΔSTP2 were enriched in the redundant downstream genes. Note that we consider the downstream genes shared by the functionally redundant genes (*e.g.*, *STP1* and *STP2*) as the redundant downstream genes. The mean change genes upon ΔSTP2 were not enriched in the downstream genes shared by *STP1/2* (Fig. 3a). This result was consistent with a previous study showing that most downstream genes of *STP2* were undetectable from single deletion of *STP2*^6^. However, mean change genes upon ΔSTP1 were enriched in the downstream genes shared by *STP1*/*2* (*p* < 0.05, Fisher's exact test; Fig. 3a). This result suggests that the deletion of *STP1* was not well compensated by *STP2*, although a previous study reported that deletion of *STP1* was compensated by *STP2*^7^. Subsequently, we quantified noise considering heterogeneity due to cell cycle. Briefly, we clustered WT and mutant cells simultaneously by similar expression pattern and executed comparison within each clusters (Fig. S1, S2 and see Material and methods). Because this analysis is vulnerable to the clustering methods, we keep this analysis as supplement. When we executed the same analysis with Fig. 3a but with considering cell cycle heterogeneity (Fig. S4a and see Materials and Methods), the same tendency with Fig. 3a were maintained. On the contrary, the noise-only change genes were enriched in the downstream genes shared by *STP1*/2 in both ΔSTP1 and ΔSTP2 (*p* < 0.05, Fisher's exact test; Fig. 4a). This result supports our hypothesis that the downstream genes of functionally redundant genes show noise-only change. When we eliminated cell cycle heterogeneity, the enrichment of noise-only change genes to common downstream genes of *STP*1/2 disappeared in ΔSTP1, but a relatively high proportion of noise-only change genes (2/12) were common downstream genes of *STP1*/2 (Fig. S5a). Note that in the above results, we treated with the downstream genes shared by the *STP*1/2 as the redundant targets and executed an enrichment test toward those genes. From a more conservative perspective, it is possible to consider the redundant targets as the downstream genes detected only from the experiment using the double deletion, ΔSTP1ΔSTP2. We executed the analysis based on this consideration regarding redundant targets. The results are shown in the Fig. S3. The same tendency as those obtained in the main results were observed for ΔSTP2, but the significant enrichment of the noise-only change for ΔSTP1 was not observed. Since in this analysis many of the true redundant genes appeared to be missing among the conservatively defined redundant genes, the consistent results regarding ΔSTP2 suggests that these findings are highly reliable.

### Non redundantly regulated target; *RTG1* and *RTG3*

Next, as control, we investigated downstream genes shared by non-redundant genes. *RTG1* and *RTG3* are not a paralog pair, but they have significant overlap in downstream genes to form a complex^6^. We investigated whether mean change genes or noise-only change genes were enriched in the downstream genes shared by *RTG1/3*. The mean change genes were enriched in the common downstream genes in both ΔRTG1 and ΔRTG3 (*p* < 0.05, Fisher's exact test; Fig. 3b). The results are reasonable because *RTG1* and *RTG3* do not have functional compensation and the perturbation of even one gene produces downstream effects. We observed the same trend even when we considered cell cycle heterogeneity (Fig. S4b). The noise-only change genes were not enriched in ΔRTG1 nor ΔRTG3 (Fig. 4b). This is also consistent with the result considering cell cycle heterogeneity (Fig. S5b). These results suggest that *RTG1* and *RTG3* do not exhibit functional compensation.

### Possibly redundantly regulated target; GATA family

Next, we focused on the GATA family, *GAT1, GZF3*, *DAL80*, and *GLN3*^12^, and investigated networks constituted from homologous gene groups. They are paralogs and possibly have redundant functions. We investigated whether GATA family members display functional compensation by estimating whether noise-only change genes are enriched in common downstream genes of the paralogous family. In ΔGZF3, the mean change genes were enriched in downstream genes shared by *GZF3* and other GATA family members (*p* < 0.05, Fisher's exact test; Fig. 3c). However, other deletion strains from the GATA family did not show enrichment of mean change genes compared to common downstream genes. When we considered cell cycle heterogeneity, any genes did not show the enrichment (Fig. S4c). The noise-only change genes were enriched in the common downstream genes of the GATA family only in ΔGLN3 (*p* < 0.05, Fisher's exact test; Fig. 4c). Although a redundant pathway has not been reported between *GLN3* and other genes, double deletion of *GLN3* and *GAT1* or *GLN3* and *DAL80* decreased growth rate^13,14^. These results suggest the existence of functional compensation between *GLN3* and *GAT1* and/or *GLN3* and *DAL80*. In Fig. S5c, any strains did not show enrichment with common downstream genes with the GATA family even when we considered cell cycle heterogeneity. The small number of common downstream genes might hinder the estimation of downstream genes.

In summary, we found that downstream genes shared by a functional compensative pair tend to show noise-only changes when one of the functional compensative pairs, such as *STP1* and *STP2,* is deleted. Although other functional compensative pairs should be used to reinforce our results, no suitable scRNA-seq data are available to perform additional examinations. scRNA-seq of yeast is still an advancing technology and the data we require is expected to accumulate from now on.

**Figure 5:**
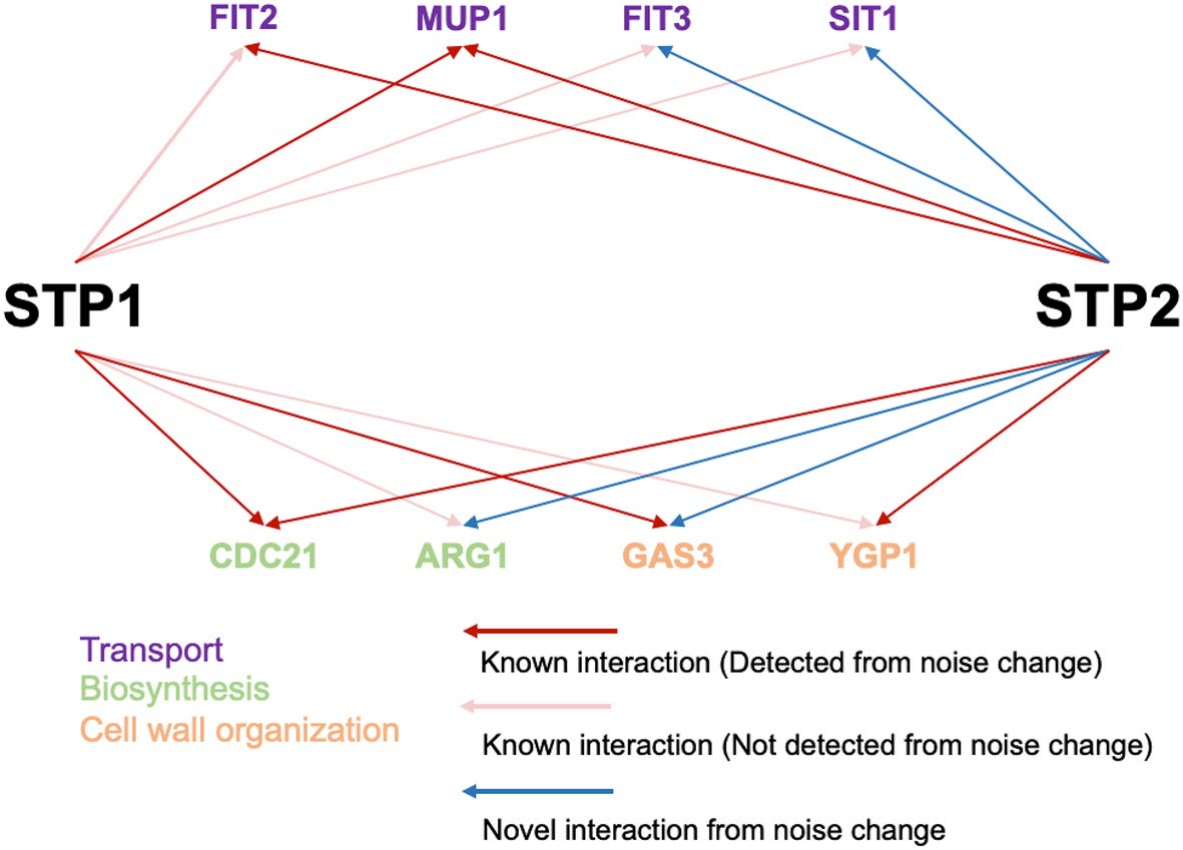
Novel and known interactions shared by STP1 and STP2. Red and blue arrows imply known and novel interactions, respectively. Light red arrows represent known interactions that cannot be detected from noise changes in this study. The color of gene names implies GO terms retrieved from SGD GO Slim mapper.

From the above results, we suggest that noise-only change captures redundantly regulated genes. Based on our concepts, the candidate of redundantly regulated genes, which was detected by noise-only change, must be common to the deletion strain of the redundant gene pair. In fact, we detected three genes (*MUP1*, *CDC21*, *GAS3*) that show noise-only change in both ΔSTP1 and ΔSTP2 (genes indicated by two dark red or blue arrows in Fig. 5), while two of these (*MUP1, CDC21*) are known as shared downstream genes of *STP1* and *STP2* (genes indicated by two dark red arrows in Fig. 5). We propose that *GAS3* is a new redundantly regulated candidate of *STP*1/2. Although an interaction between *STP2* and *GAS3* has not been reported, an interaction between *STP1* and *GAS3* was reported^6^. This supports that *GAS3* is a new redundantly regulated candidate by *STP*1/2. Notably, although the number of noise-only change genes common to ΔSTP1 and ΔSTP2 was three, the number of mean change genes common to ΔSTP1 and ΔSTP2 was zero. These results support our hypothesis that redundantly regulated genes that cannot be detected from mean change but are detected from noise-only change.

However, not all noise-only change genes are common to ΔSTP1 and ΔSTP2. The total number of noise-only change genes in ΔSTP1 is 15, while there are eight in ΔSTP2. As described above, the three genes overlap. Most noise-only change genes are detected only in ΔSTP1 or ΔSTP2. We speculate that this asymmetric noise-only change is caused by (1) artifact of FDR threshold, (2) asymmetric compensation, or (3) compensation by other genes. First, since the extent of noise difference caused by gene deletion is attributed to physical properties of the deleted gene, such as translational efficiency^9^, the threshold must differ gene by gene. Applying the same cut off to all genes leads to increased false negative results. The precise modeling of noise propagation must improve detection of redundant regulation. Second, asymmetric functional compensation might be considered. For example, the deletion of *STP2* is compensated by *STP1*, but the deletion of *STP1* is not compensated by STP2 with fewer interactions. In such situations, the gene shows mean change upon ΔSTP1 and on the contrary the gene shows noise-only change upon ΔSTP2. Third, functional compensation by other genes might be considered. We assumed that functional compensation is likely to occur between paralogous pairs due to their functional similarity, but there are many synthetic effects between *STP1/2* and other genes^13^. However, a synthetic effect does not necessarily translate to functional compensation, but those interactions suggest that a lack of *STP1* or *STP2* functionality may possibly be compensated by the other genes.

### Novel downstream candidates of *STP1* and *STP2* detected from noise change

We investigated noise change genes, including mean change, to determine whether noise difference tests narrow down the reasonable downstream genes. We hypothesized that noise-only change genes shared by the deletion strain of a paralogous pair, such as ΔSTP1 and ΔSTP2, are likely to be the target of functional compensation. However, the noise change genes accompanied with mean change genes also can be recognized as downstream gene candidates. Interestingly, both known *STP2* downstream genes and novel *STP2* downstream candidates detected from noise change were enriched in transport-related genes (*p* < 0.01; SGD GO Term Finder; Process; Table 1). Therefore, it can be argued that the noise difference test has targeted reasonable candidates.

**Table 1:** Noise-change genes in ΔSTP1 and ΔSTP2.

Name	Interaction	GO term
**STP1**		
BIO2	Novel	Vitamin metabolic process, monocarboxylic acid metabolic process
RPL26A	Novel	Cytoplasmic translation, ribosomal large subunit biogenesis
ILV5	Novel	Cellular amino acid metabolic process, mitochondrion organization
CLN1	Novel	Protein phosphorylation, mitotic cell cycle, regulation of protein modification process, regulation of cell cycle
WSC2	Novel	Cell wall organization or biogenesis, response to heat
MET6	Known	Cellular amino acid metabolic process
MUP1	Known	Ion transport, amino acid transport, transmembrane transport
TOS4	Known	Cellular response to DNA damage stimulus
GAS3	Known	Cell wall organization or biogenesis, carbohydrate metabolic process
CDC21	Known	Nucleobase-containing small molecule metabolic process
**STP2**		
TIM9	Novel	Mitochondrion organization, protein targeting
SIT1	Novel	Ion transport, transmembrane transport, cellular ion homeostasis
NOP16	Novel	rRNA processing, ribosomal large subunit biogenesis
HXT4	Novel	Ion transport, transmembrane transport, carbohydrate transport
GSY2	Novel	Carbohydrate metabolic process, generation of precursor metabolites and energy
RPS17A	Novel	Ribosomal small subunit biogenesis, ribosome assembly, cytoplasmic translation
GAS3	Novel	Cell wall organization or biogenesis, carbohydrate metabolic process
ARG1	Novel	Cellular amino acid metabolic process
FIT3	Novel	Ion transport
CIN2	Novel	Protein folding, cell morphogenesis
CLN2	Novel	Mitotic cell cycle, protein phosphorylation, response to chemical, regulation of cell cycle, regulation of protein modification process, conjugation
MUP1	Known	Ion transport, transmembrane transport, amino acid transport
YGP1	Known	Cell wall organization or biogenesis
CDC21	Known	Nucleobase-containing small molecule metabolic process
FIT2	Known	Ion transport

Name: Genes that showed noise change in ΔSTP1 or ΔSTP2 are listed; Interaction: Noise change genes that had not been reported as STP1 or STP2 downstream in Yeastract are labeled as “Novel”; GO term: GO terms retrieved from SGD GO slim mapper (Yeast GO-Slim process). In the novel STP1 candidates, there were no enriched GO terms (SGD GO term finder process). In the novel STP2 candidates, GO terms were enriched in transport-related genes, in which STP2 downstream genes are involved (*p* < 0.01; SGD GO Term Finder; Process).

In this study, we executed two types of analysis. (1) The conservative difference test, which quantified values from all cells (Figs. 3 and 4), and (2) the progressive difference test, which quantified values from cells belonging to the same cluster (Fig. S4 and S5). In the later progressive analysis, as cell cycle heterogeneity is excluded from noise quantification, we can quantify that the noise change originated from a molecular process (but the results are affected by clustering). In the former conservative analysis, *CLN1*, which is related to cell cycle regulation and does not interact with *STP1*, showed noise change upon ΔSTP1, but in the later progressive analysis, *CLN1* did not show noise change upon ΔSTP1 (Table S2). In the same way, *CLN2*, which is related to the cell cycle and does not interact with *STP2*, showed noise change upon ΔSTP2, but when we consider cell cycle heterogeneity, *CLN2* did not show significant noise change (Table S2). This implies that the noise change of *CLN1* and *CLN2* originated from differences in cell cycle heterogeneity that are out of our interests. We consider that the consistent results of conservative and progressive analysis should be reliable. We found four consistent genes in ΔSTP2 that show noise change, both from conservative and progressive analysis (*CDC21, SIT1, FIT2, FIT3*). *FIT2* and *CDC21* are known as shared downstream genes of *STP1* and *STP2* (genes indicated by two dark red arrows in Fig. 5). *SIT1* and *FIT3* are possibly newly downstream candidates of *STP2*, and function as transporters, similar to the many known downstream genes of *STP2*. In addition, *SIT1* and *FIT3* are known as downstream genes of *STP1* (genes indicated by dark red arrows and blue arrows in Fig. 5). This suggests they are redundantly regulated by *STP1* and *STP2*. Note that *SIT1* and *FIT3* are the noise-only change genes.

In summary, we detected four novel gene candidates that are redundantly regulated by *STP1* and *STP2* (*ARG1, FIT3, SIT1, GAS3*), even from the single deletion strain of *STP2* (Genes indicated by red arrow and blue arrow in Fig. 5). Three of these (*FIT3, SIT1, GAS3*) are constant, as we observed consistent results across progressive and conservative analyses. Interestingly, *ARG1* is involved in the arginine biosynthesis process^15^, and it was previously unknown that *STP2* affects this process. Thus, our results suggest that *STP2* may be involved in the arginine biosynthesis process.

Based on the above results, we constructed the gene interaction network shared by *STP1* and *STP2* (Fig. 5). In the previous study, *MUP1*, *FIT2*, and *YGP1* were only detected from mean change in the double deletion of *STP1* and *STP2*^4,5^. Namely, they cannot be detected in single deletions of *STP1* or *STP2*. However, in this study, those hidden interactions could be detected by the noise-only change, even from the single deletion of *STP2*. In addition, several new *STP2* downstream genes were detected by noise change. We found that some of these were also downstream genes of *STP1*, which supports that they are redundantly regulated by *STP*1/2 (genes indicated by blue and red arrow in Fig. 5). Here, we discuss about the reason why those novel *STP*2 downstream genes that have redundant interaction with *STP1*, had not been found by ΔSTP1ΔSTP2 nor ΔSTP2 in the previous study. If the effect of *STP1* is much larger than that of *STP2* in redundant pathway, the mean expression change of downstream genes estimated in ΔSTP1ΔSTP2 may not differ from that in ΔSTP1 due to the subtle effect of *STP2*. Namely, the detection of asymmetric functional compensation is difficult, even from the double deletion, unless we use noise-only change.

Conventionally, redundantly regulated genes are detected by assessing mean expression change in the double deletion of redundant genes, in order to avoid functional compensation. From this analysis, we propose that redundantly regulated genes can be detected by assessing noise-only change, even in the single deletion of one of the redundant genes. Our new approach will provide a novel framework for revealing complex gene networks. As a limitation of this study, because it is necessary to quantify the variation of expression levels in homogeneous cells, as we showed in this study (Fig. S1 and see Materials and Methods), its application is limited to unicellular organisms at this stage. Furthermore, the quantified noise includes intrinsic noise and extrinsic noise, which later one is not our interests. More accurate interaction analysis should be possible by eliminating extrinsic noise and focusing on intrinsic noise. In this study, we roughly excluded extrinsic noise by clustering scRNA-seq data (Fig. S1, S2). We propose that exact modeling of extrinsic and intrinsic noise using scRNA-seq data will allow for more accurate noise difference estimation. We consider that noise difference tests can be applied as a new strategy for data-driven network estimation that can capture even functional compensation.

## Materials and methods

### Expression data

We used single-cell RNA-seq data in yeast, including eight different TF deletion strains (ΔSTP1, ΔSTP2, ΔRTG1, ΔRTG3, ΔGLN3, ΔGAT1, ΔGZF3, and ΔDAL80) and wild type (WT) as a control^16^. ΔSTP1 and ΔSTP2 were used to investigate a redundant gene pair. ΔRTG1 and ΔRTG3 were used to investigate a non-redundant gene pair that shares many target genes. ΔGAT1, ΔGZF3, ΔDAL80, and ΔGLN3 were used to investigate paralogues gene groups, from which redundant functions have not been reported. Each strain had six biological replicates, which were independently constructed and cultured under YPD conditions. Six of the deleted TFs were paralogs (BLASTP search, E-value < 10^–10^) and two of these were singletons (Table S1). We obtained transcript counts from individual cells, which passed quality control, followed by removal of doublets according to Jackson et al.^16^.

### Elimination of cell cycle heterogeneity

In order to precisely quantify the changes in expression noise caused by gene deletion, cell heterogeneity due to cell cycle should be eliminated. Therefore, we divided cells into groups exhibiting similar expression patterns. Two comparative strains were clustered simultaneously. This enabled us to compare two strains within a cluster (Fig. S1). In order to cluster cells by their expression state, first, we normalized cell specific biases using scran normalization methods^17^. Subsequently, we constructed shared nearest neighbor graphs^18^ from normalized counts, and clustered these using Louvain methods^19^. As a result, the strains were grouped into five or six clusters (Fig. S2). Although cells of two strains were randomly distributed in the clusters, expression patterns of *DSE2*^20^, *PIR1*^21^, and *HTB*^22^, known as cell cycle marker genes, were biased by clusters (Fig. S2). Thus, cells were thought to be clustered by cell cycle. Since each cluster represents a specific cell state, two strain comparisons within a cluster was not thought to be affected by heterogeneity due to cell cycle. Since the comparison was executed by cluster, noise (mean) difference results for one gene in a strain equated to the number of clusters. If a gene showed significant change (FDR < 0.01; Bonferroni correction) in at least one cluster, we considered the gene as a downstream gene candidate of the deleted gene. The results were shown in Fig. S4 and S5. Note that clustering cells based on cell cycle requires a large sample size (number of cells) because the expression pattern in a sampled population is desired to reflect the true populational distribution. Since the sample size of ΔGLN3 is small, clustering analysis was not executed in that strain. Detected genes in this method are not robust as they are affected by clustering.

### Filtration of technical noise

As we described above, each strain includes six biological replicates constructed from different experiments. Note that variance between replicates is smaller than the variance between compared strains (Fig. S6). These replicates were utilized to estimate technical noise under the assumption in which replicate specific variation is technical artifact, and common variation between replicates is biological noise (Fig. 2a; panel 2). Further, Poisson noise is subtracted from the variance as it is interpreted as the RNA sampling noise of the sequencer. As a result, over-dispersion of Poisson noise after accounting for technical noise is considered as biological noise. The example of variance decomposition is shown in the Fig. S6. This filtration was executed using the hierarchical Bayesian model in BASiCS R package^10,11^.

### Expression level comparison

To test the hypothesis that functional compensation is not detected by mean change but can be detected by noise change, we compared differences in mean expression levels for each gene between wildtype and deletion mutant with differences in expression noise (Fig. 2a). The mean expression level and expression noise were estimated using the hierarchical Bayesian model in BASiCS R package^10,11^. All parameters of MCMC were set to the recommended values in the BASiCS R package manual. Since the expression noise used in the comparison was a residual measure of variability that is not correlated with mean expression level, expression noise change is independent from mean change. The details were reported in Eling et al.^10^ (residual over-dispersion). After comparing the mean with noise respectively, genes showing a significant change with EFDR less than 0.01 were estimated as downstream genes of the deleted gene.

### Validation of estimation results

To investigate whether estimated downstream genes were enriched in reported downstream genes, we used the experimentally confirmed interaction database, Yeastract^23^. We retrieved the regulation matrix from Yeastract. Only expression evidence detected under unstressed log-phase growth conditions were included.

### Criteria for false negative and false positive targets

In the mean or noise difference tests regarding expression levels, we referred to the false negative target and false positive target. These identifications are based on the Yeastract data which we used as a gold standard. False negative targets are recognized as genes that we could not estimate but have been reported on the Yeastract. On the contrary, genes that we could detect but have not been reported on the Yeastract are possibly novel candidates of downstream genes. However, the newly detected genes that are not related to the known downstream genes or GO terms of known downstream genes are recognized as false positive targets.

## Supplementary Information

Supplementary Information.

## Data Availability

All data analyzed during this study are included in this manuscript. Scripts used in this analysis have been deposited in zenodo (session ID: 5041324).

## Abbreviations

GRN: Gene regulatory network
TF: Transcription factor
