# Supplementary material for: Capturing hidden regulation based on noise change of gene expression level from single cell RNA-seq in yeast
Source: Sci Rep. 2021 Nov 19;11:22547. doi: 10.1038/s41598-021-01558-y (PMC8604932; doi:10.1038/s41598-021-01558-y)
Supplement: Supplementary file 1 — Supplementary Information. [file 41598_2021_1558_MOESM1_ESM.pdf]

Table S1. Eight deletion strains and associated paralogs.

Strain	Paralog
ΔSTP1	STP2
ΔSTP2	STP1
ΔDAL80	GLN3, GAT1, GZF3
ΔGLN3	DAL80, GAT1, GZF3
ΔGAT1	DAL80, GLN3, GZF3
ΔGZF3	DAL80, GLN3, GAT1
ΔRTG1	Nothing
ΔRTG3	Nothing

Table S2. Noise-change genes in ΔSTP1 and ΔSTP2 considering cell cycle heterogeneity.
Name: Genes that showed noise change considering cell cycle heterogeneity are listed; Interaction: Noise change genes that had not been reported as STP1 or STP2 downstream in Yeastract are labeled as "Novel"; GO terms: GO term retrieved from SGD GO slim mapper (Yeast GO-Slim process). In novel STP1 candidates, there were no enriched GO terms (SGD GO term finder process). In novel STP2 candidates, GO terms were enriched in transport-related genes, in which STP2 downstream genes are involved ($p < 0.01$; SGD GO Term Finder; Process).

Name	Interaction	GO term
STP1		
RGI1	Novel	generation of precursor metabolites and energy
DSE2	Novel	pseudohyphal growth
HSP150	Novel	cell wall organization or biogenesis
ASH1	Novel	regulation of cell cycle, pseudohyphal growth, mitotic cell cycle, transcription by RNA polymerase II
MMR1	Novel	mitochondrion organization, organelle inheritance
FRE1	Novel	ion transport, cellular ion homeostasis
AIM44	Novel	regulation of cell cycle, mitotic cell cycle, regulation of organelle organization, cytoskeleton organization, cytokinesis
HSP82	Novel	mitochondrion organization, regulation of organelle organization, protein targeting, regulation of DNA metabolic process, telomere organization, response to osmotic stress, response to heat, protein maturation, protein folding
HXT3	Known	ion transport, transmembrane transport, carbohydrate transport
MET6	Known	cellular amino acid metabolic process
ZRT1	Known	ion transport, transmembrane transport
FIT2	Known	ion transport
STP2		
SIT1	Novel	ion transport, transmembrane transport, cellular ion homeostasis
SCW11	Novel	carbohydrate metabolic process, cell wall organization or biogenesis
PRY1	Novel	lipid transport
PIR1	Novel	cell wall organization or biogenesis
AHP1	Novel	response to chemical, response to oxidative stress
CTS1	Novel	carbohydrate metabolic process
MET17	Novel	cellular amino acid metabolic process
PHO89	Known	ion transport, transmembrane transport
PRY3	Known	lipid transport, conjugation
PCL1	Known	regulation of protein modification process, regulation of cell cycle, protein phosphorylation, cytoskeleton organization
CDC21	Known	nucleobase-containing small molecule metabolic process

Figure S1. Schematic workflow for the elimination of cell cycle heterogeneity. The upstream workflow of this step corresponds to steps 1~3 in Figure 2. The steps shown in this figure were only executed in the progressive analysis in the supplement. 1) To eliminate cell cycle heterogeneity, we clustered the wildtype and strain of interest. It is already known that the difference of expression pattern of two strains is smaller than the difference of cell heterogeneity within the strain. Thus, the two strains are clustered into the same cluster by their cell cycle state. 2) After we confirmed that the distribution of the two strains were not biased in a cluster, the two strains were compared within the cluster in the expression mean and noise. 3) Consequently, we achieved as many results from the mean and noise difference tests as the number of clusters. These are consolidated into one by interpreting the significant change genes as the genes that showed significant change in at least one cluster. From the consolidated list, we extracted mean change genes and noise-only change genes. The downstream workflow corresponds to step 5 in Figure 2.

Figure S2. Cells clustered by cell cycle state. Dimension composition was conducted using UMAP. First row: Clustering results; Second row: genotype mapping. Yellow and green plots represent mutant deletion and wildtype, respectively; Third row: *PIR1* expression pattern; Fourth row: *DSE2* expression pattern; Fifth row: *HTB* expression pattern.

Figure S3. Ratio of mean and noise-only change genes in known downstream genes detected from double deletion ΔSTP1ΔSTP2. a) Bars show the proportion of mean change genes in the two categories labeled in the x-axis. Genes detected in ΔSTP1ΔSTP2; The reported downstream genes detected from the double deletion strain, ΔSTP1ΔSTP2. Others; the other genes in the right category. Bars show the ratio of mean change genes in each category. Panels on the left and right show the result of mean change genes in ΔSTP1 and ΔSTP2, respectively. b) Bars show the proportion of noise-only change genes in the two categories labeled in the x-axis. Genes detected in ΔSTP1ΔSTP2; The reported downstream genes detected from the double deletion strain, ΔSTP1ΔSTP2. Others; the other genes in the right category. Bars show the ratio of noise-only change genes in each category. Panels on the left and right show the result of noise-only change genes in ΔSTP1 and ΔSTP2, respectively. Note that the numbers of genes detected in ΔSTP1ΔSTP2 (shown on the red bar) in ΔSTP1 (left) and ΔSTP2 (right) do not coincide because non expressed genes in each strain are excluded.

Figure S4. Ratio of mean change genes detected after eliminating the cell cycle effect in known downstream genes shared by homologous groups. The lists of mean change genes derived from some clusters (cell cycle state) are consolidated into one list by interpreting the significant change genes as the genes that showed mean change in at least one cluster (FDR < 0.01; Bonferroni correction). The representation of this figure is the same as in Figure 3.

Figure S5. Ratio of noise-only change genes detected after eliminate cell cycle effect in known downstream genes shared by homologous groups. The lists of noise-only change genes derived from some clusters (cell cycle state) are consolidated into one list by interpreting the significant change genes as the genes that showed noise-only change in at least one cluster (FDR < 0.01; Bonferroni correction). The representation of this figure is the same as in Figure 4.

Figure S6. Comparison between replicates in wildtype and ΔSTP1. Expression data of *ILV5* are shown as an example of a high expression gene. a) Distribution of *ILV5* expression level by replicates in each of wildtype(a1) and ΔSTP1(a2). The x-axis shows the read count and the y-axis shows the number of cells. Note that we did not analyze the variance derived from the raw distribution. Rather, we analyzed the biological variance which eliminates the technical artifact as shown in the panel b. b) Variance of *ILV5* expression level decomposed into the non-biological noise (technical and shot noise) and biological noise by replicates in each of wildtype(b1) and Δ STP1(b2). The y- and x-axes show the percentage and replica id, respectively. Biological: variance explained by a biological factor. Technical: variance explained by technical factor originated in the experiment. Shot noise: sampling noise of RNA caused in the sequencer.

Figure S1

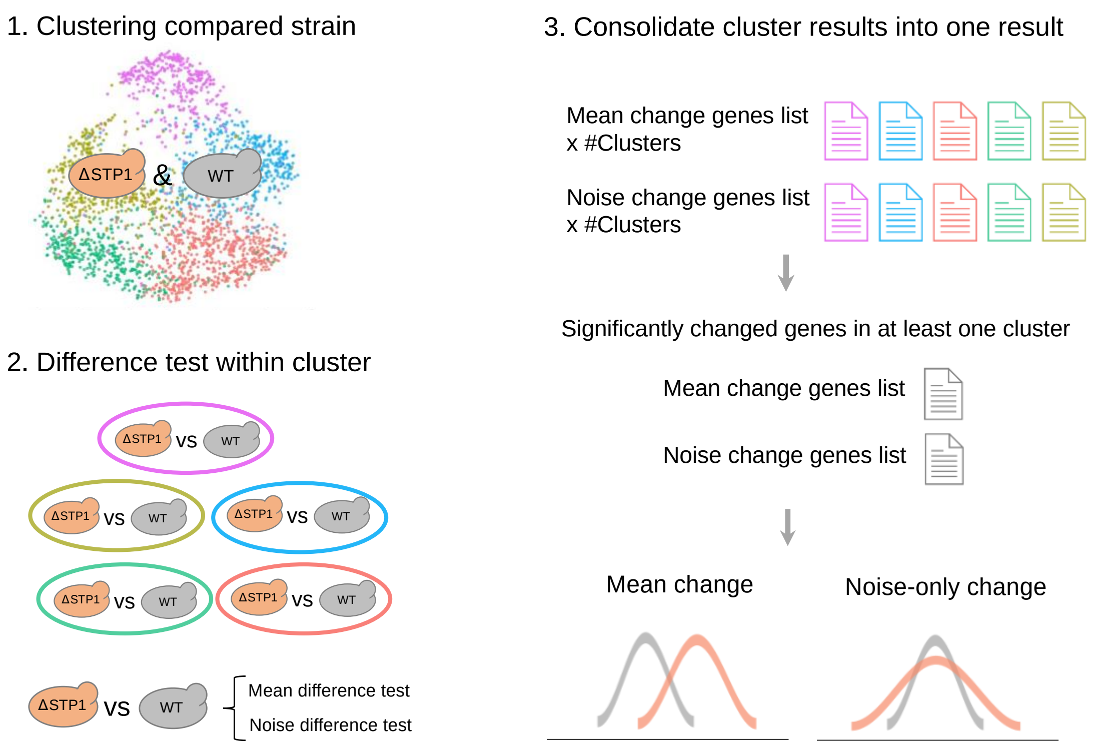
1. Clustering compared strain
ΔSTP1
&
WT
2. Difference test within cluster
ΔSTP1
vs
WT
Mean difference test
Noise difference test
3. Consolidate cluster results into one result
Mean change genes list
x #Clusters
Noise change genes list
x #Clusters
Significantly changed genes in at least one cluster
Mean change genes list
Noise change genes list
Mean change
Noise-only change

Figure S2

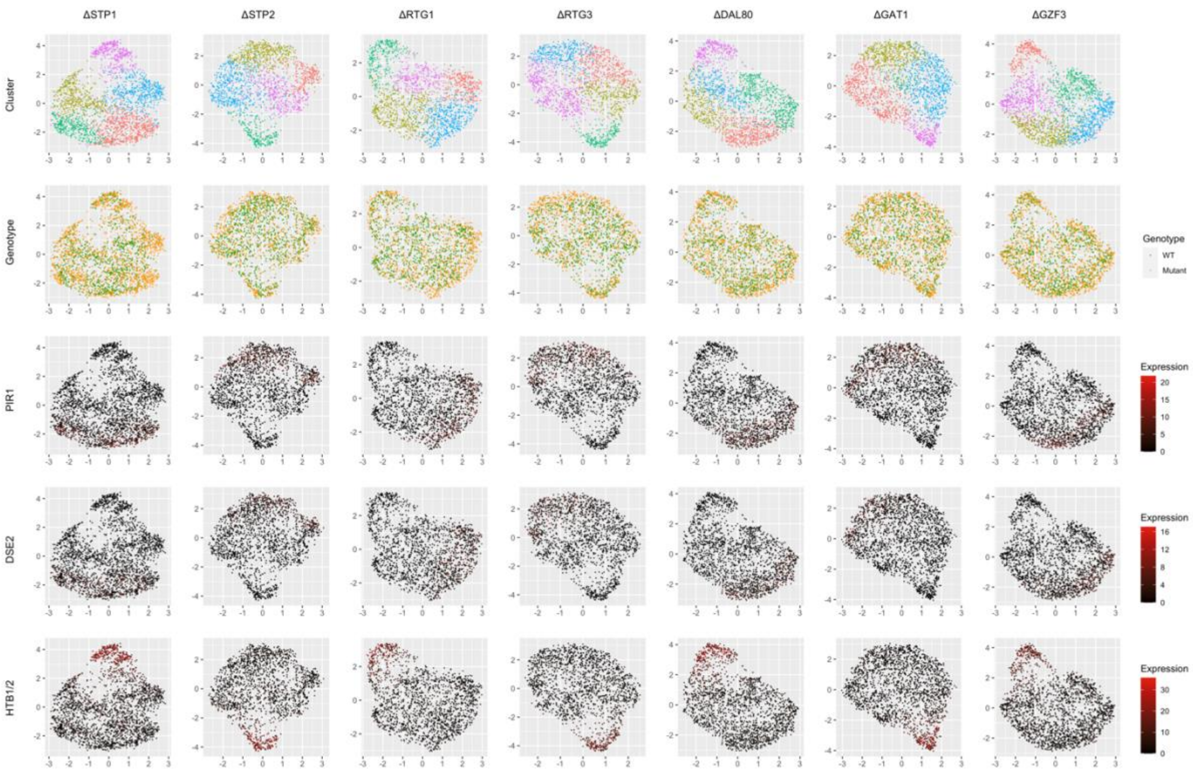

ΔSTP1
ΔSTP2
ΔRTG1
ΔRTG3
ΔDAL80
ΔGAT1
ΔGZF3
Cluster
Genotype
PIR1
DSE2
HTB1/2
Genotype
WT
Mutant
Expression

Figure S3

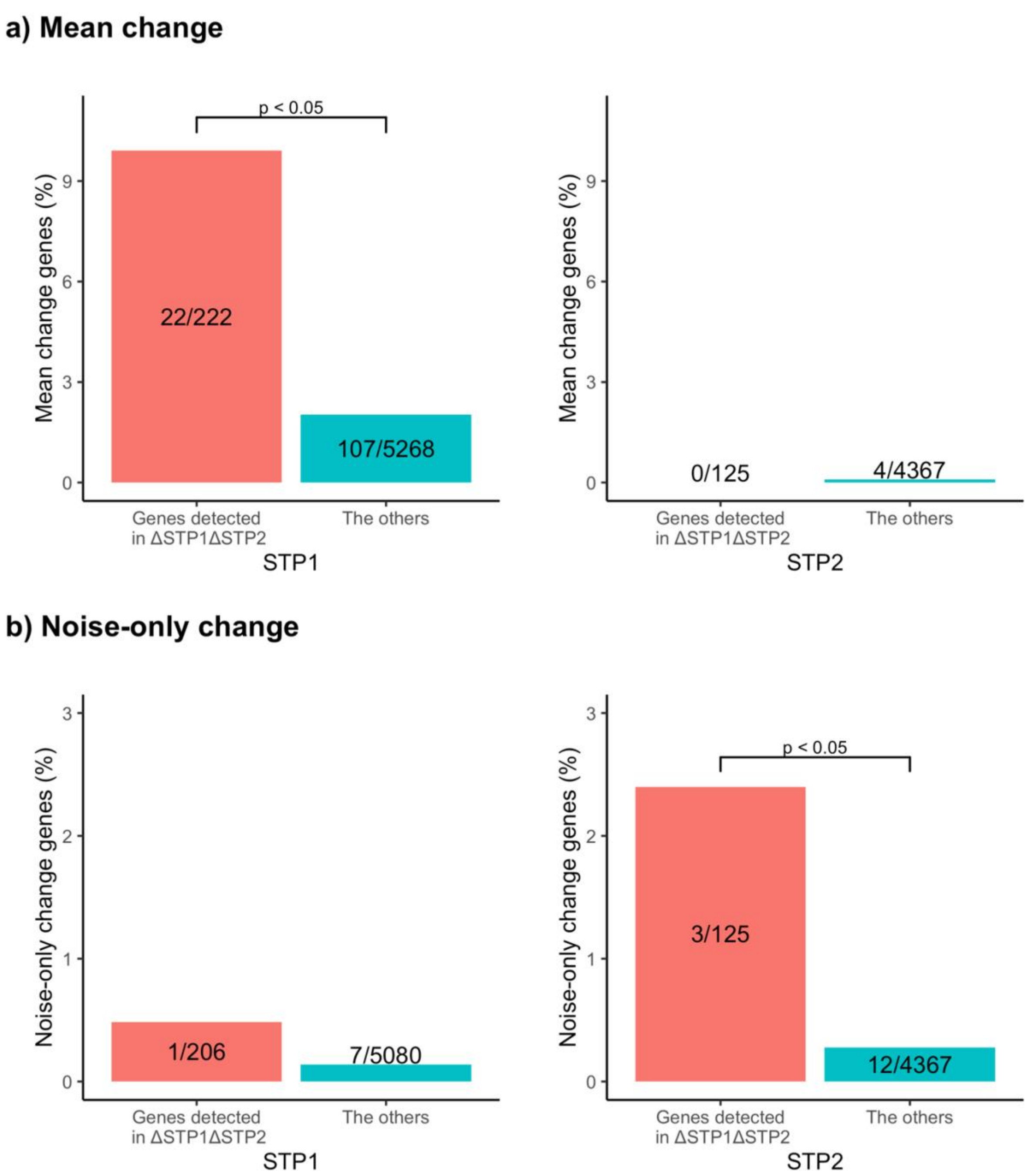
a) Mean change
p < 0.05
Mean change genes (%)
9
6
3
0
22/222
107/5268
Genes detected
in ΔSTP1ΔSTP2
The others
STP1
Mean change genes (%)
0/125
4/4367
STP2
b) Noise-only change
Noise-only change genes (%)
3
2
1
0
1/206
7/5080
Genes detected
in ΔSTP1ΔSTP2
The others
STP1
p < 0.05
3/125
12/4367
STP2

Figure S4

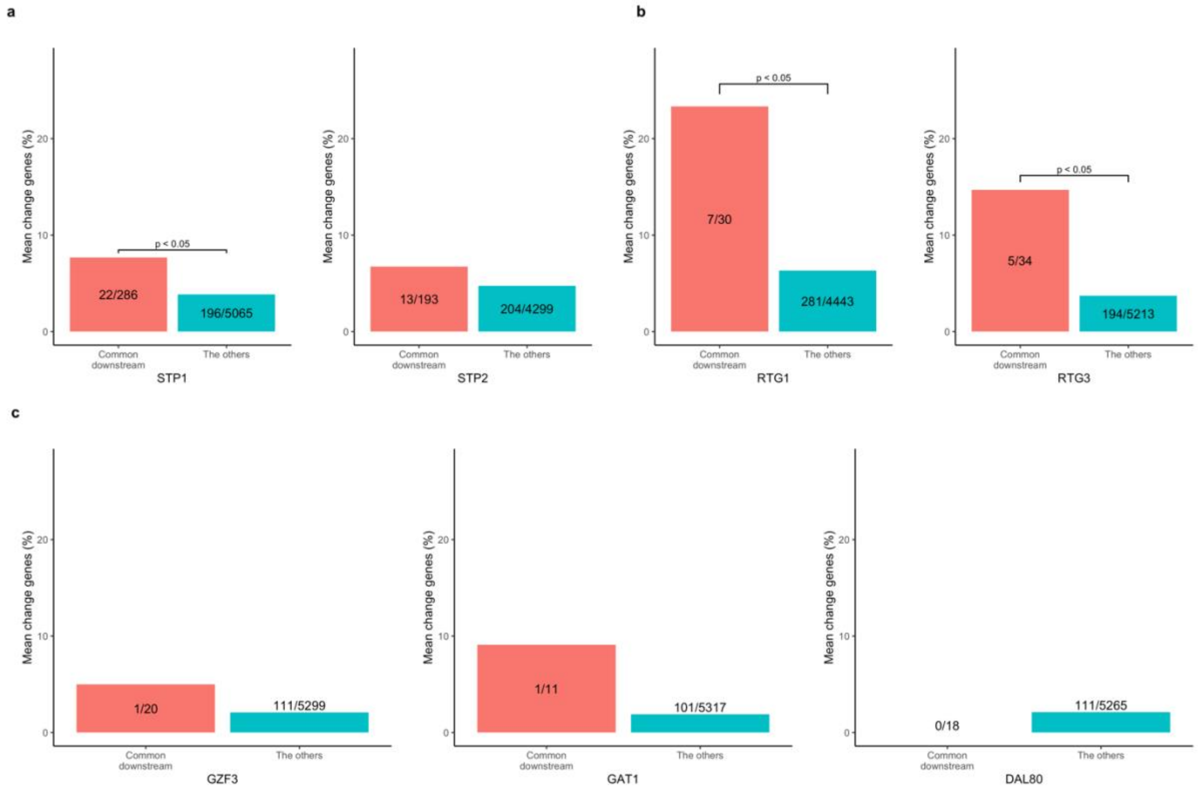
a
b
c
Mean change genes (%)
p < 0.05
22/286
196/5065
13/193
204/4299
7/30
281/4443
5/34
194/5213
Common downstream
The others
STP1
STP2
RTG1
RTG3
1/20
111/5299
1/11
101/5317
0/18
111/5265
GZF3
GAT1
DAL80

Figure S5

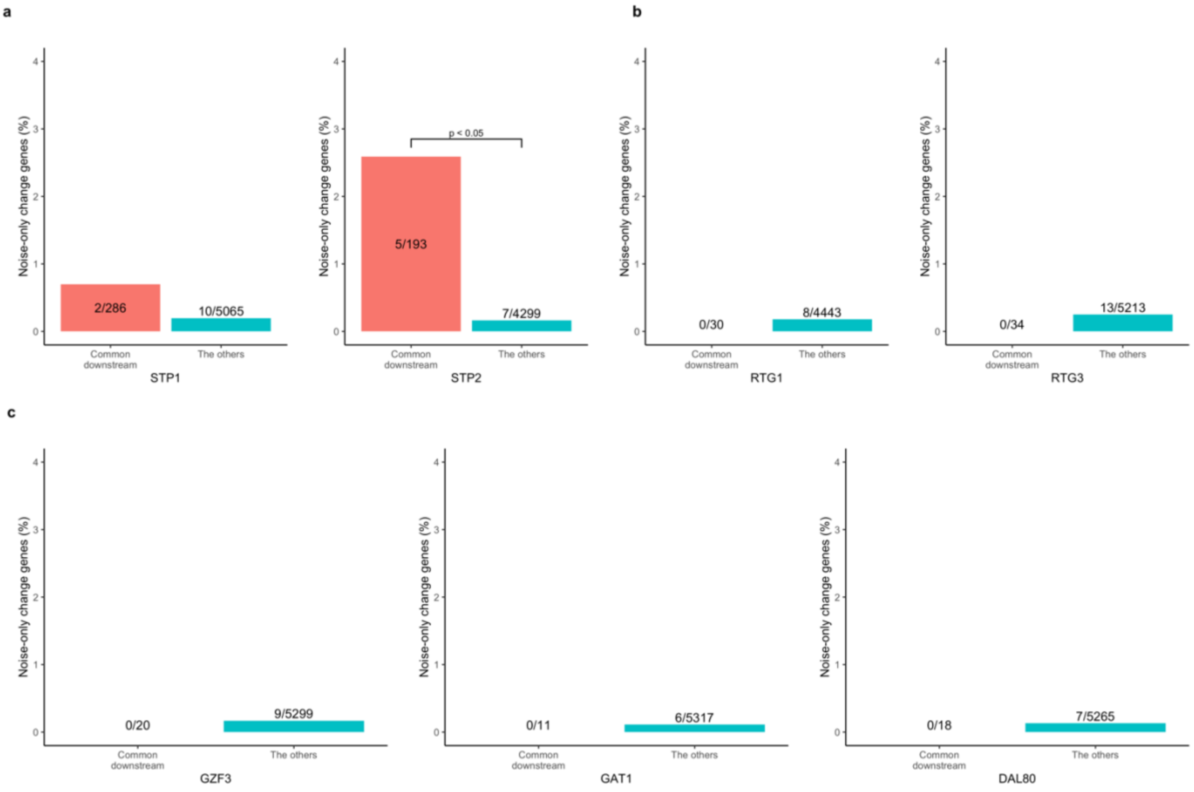
a
b
c
Noise-only change genes (%)
2/286
10/5065
Common downstream
The others
STP1
p < 0.05
5/193
7/4299
STP2
0/30
8/4443
RTG1
0/34
13/5213
RTG3
0/20
9/5299
GZF3
0/11
6/5317
GAT1
0/18
7/5265
DAL80

Figure S6

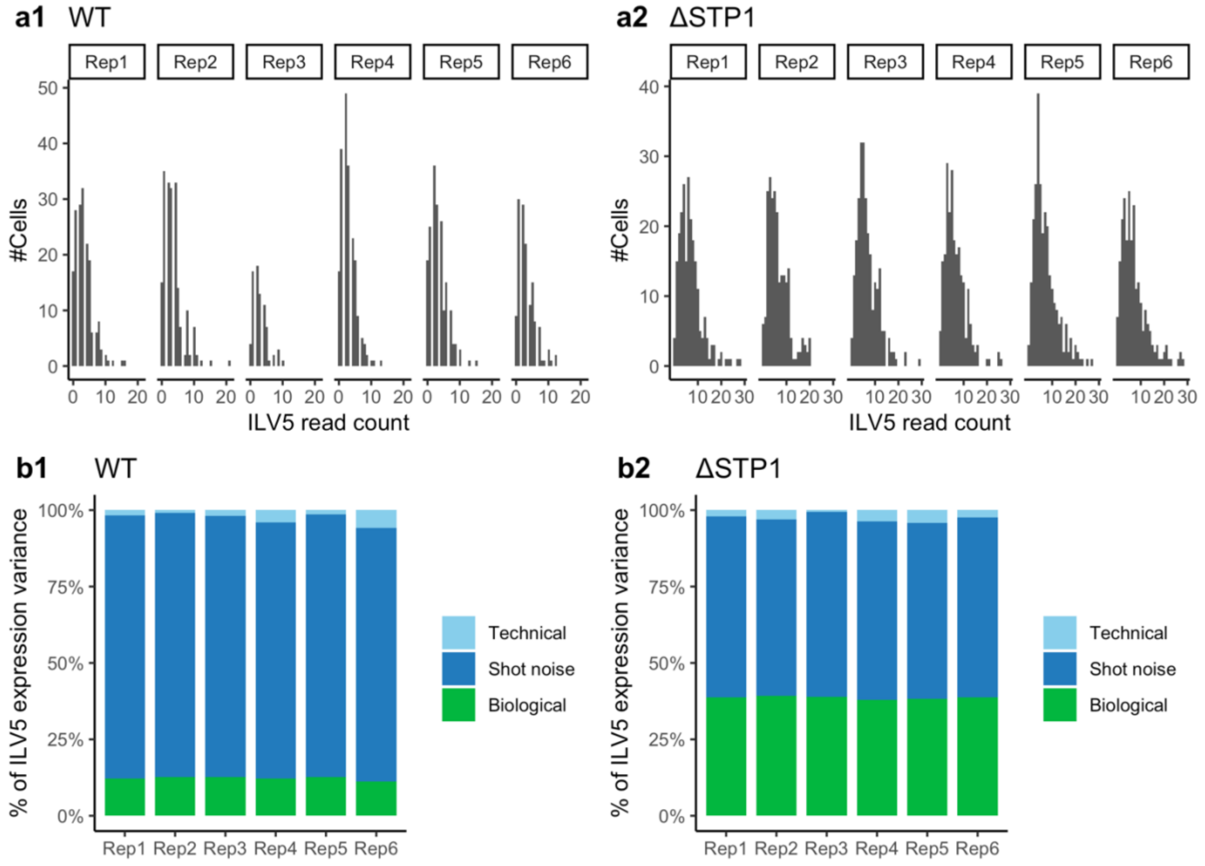
a1 WT
Rep1
Rep2
Rep3
Rep4
Rep5
Rep6
#Cells
ILV5 read count
a2 ΔSTP1
b1 WT
% of ILV5 expression variance
Technical
Shot noise
Biological
b2 ΔSTP1
